# Effects of *Citrus kawachiensis* Peel in Frailty-like Model Mice Induced by Low Protein Nutrition Disorders

**DOI:** 10.3390/antiox12030779

**Published:** 2023-03-22

**Authors:** Toshiki Omasa, Satoshi Okuyama, Atsushi Sawamoto, Mitsunari Nakajima, Yoshiko Furukawa

**Affiliations:** Department of Pharmaceutical Pharmacology, College of Pharmaceutical Sciences, Matsuyama University, 4-2 Bunkyo-cho, Matsuyama 790-8578, Japan

**Keywords:** *Citrus kawachiensis*, frailty, microglia, brain

## Abstract

“Frailty” caused by a decline in physiological reserve capacity, chronic inflammation, and oxidative stress in the elderly has recently become a major social issue. The present study examined the effects of the peel of *Citrus kawachiensis* (CK), which exhibits anti-inflammatory, antioxidant, and pro-neurogenesis activities in frailty-like model mice. Male C57BL/6 mice (15 weeks old) were fed an 18% protein diet (CON), a 2.5% protein diet (PM), and PM mixed with 1% dried CK peel powder for approximately 1 month. Mice were euthanized 2 or 8 days after a single intraperitoneal administration of lipopolysaccharide (LPS) and tissues were dissected. Among peripheral tissues, muscle weight, liver weight, and blood glucose levels were significantly higher in the PM–LPS–CK group than in the PM–LPS group. In the behavioral analysis, locomotive activity was significantly lower in the PM–LPS group than in the PM group. The reduction in locomotive activity in the PM–LPS–CK group was significantly smaller than that in the PM–LPS group. The quantification of microglia in the hippocampal stratum lacunosum-moleculare revealed that increases in the PM–LPS group were significantly suppressed by the dried CK peel powder. Furthermore, the quantification of synaptic vesicle membrane proteins in the hippocampal CA3 region showed down-regulated expression in the PM–LPS group, which was significantly ameliorated by the administration of the dried CK peel powder. Collectively, these results suggest that CK inhibits inflammation and oxidative stress induced by PM and LPS in the central nervous system and peripheral tissue. Therefore, *C. kawachiensis* is highly effective against “frailty”.

## 1. Introduction

Lifespans have been increasing worldwide with advances in health care and better diets, and many countries are now experiencing population aging. Aging is associated with an increased risk of a few diseases due to chronic inflammation, oxidative stress, and a decline in physiological reserve capacity. Aging decreases physical activity and food intake, resulting in protein-energy malnutrition, and has a negative impact on immunity and muscle strength. An increased susceptibility to stress, chronic inflammation, oxidative stress, and other factors may lead to a decline in the activities of daily living, the need for nursing care, and even death, and this is called “frailty” [1,2,3]. The repetition of these conditions leads to a vicious cycle, referred to as the “frailty cycle”, which has become a major social issue. Frailty includes physical frailty, such as sarcopenia and locomotive syndrome, and mental and psychological frailty related to cognitive dysfunction and depression-like symptoms, both of which may exacerbate the frailty cycle and, thus, must be carefully managed [1,2,3]. Moreover, oxidative stress caused by inflammatory cytokines and free radicals due to chronic inflammation has been implicated in various chronic diseases, such as autoimmune diseases, cancer, neurological diseases, and lifestyle-related diseases [4,5,6]. It is an important factor that accelerates the frailty cycle by creating an internal environment in which compensatory mechanisms are impaired. Furthermore, the propagation of inflammation not only to the periphery but also to the central nervous system has been suggested to accelerate brain aging and is also associated with mental disorders and neurodegenerative-related diseases, including Alzheimer’s disease [7], in which neuronal damage due to microglial activation and decreased brain-derived neurotrophic factor (BDNF) production are prominent features.

Citrus fruits contain a variety of bioactive components, especially polyphenols and flavonoids. In recent years, several citrus components have been reported to have a potential to prevent or treat not only peripheral diseases, but also central nervous system degenerative diseases caused by inflammation and oxidative stress [8]. Our laboratory extracted functional components from various citrus peels and screened them using cultured neurons, and we found that the components in *Citrus kawachiensis* peel were highly active in terms of brain-protective effects [9]. Therefore, our research has been focusing on the effects of peel components of *Citrus kawachiensis*, a specialty citrus fruit in Japan [10], on the suppression of central nervous system dysfunction induced by chronic inflammation or oxidative stress. The peel of *C. kawachiensis* contains larger amounts of bioactive components, such as 3,5,6,7,8,3′,4′-heptamethoxyflavone (HMF), auraptene (AUR), and naringin (NGI), than other citrus varieties [10,11], which are expected to exert ameliorative effects on the functional decline of the central nervous system and peripheral tissue. We previously reported that *C. kawachiensis* exhibited various anti-inflammatory, antioxidant, and brain-protective activities, such as the suppression of microglial activation and reductions in glutathione levels in the brain, in global cerebral ischemia model mice [12]. It also inhibited microglial activation and tau phosphorylation and enhanced neurogenesis in the brains of hyperglycemia model mice [13] and aging-accelerated model mice [14].

A protein malnutrition model has been reported, and the experiment was conducted by feeding a low-protein diet for four weeks [15]. The model showed hippocampal protein thiols decrease with a low protein diet and dramatic reactive gliosis accompanied by extensive neuronal loss following ischemia [15]; in addition, protein malnutrition has also been reported to decrease antioxidant capacity in the brain and may lead to increased oxidative stress [16], so a low protein diet impairs functional outcomes. Lipopolysaccharide has also been reported to induce oxidative stress and exacerbate inflammation in the body in animal models [17], suggesting that protein malnutrition and lipopolysaccharide administration-induced chronic inflammation reproduce frailty in the body. In the present study, we investigated the effects of dried *C. kawachiensis* peel powder on the central nervous system and peripheral tissues of frailty-like model mice induced by low-protein nutritional disorders.

## 2. Materials and Methods

### 2.1. Animals

All animal experiments were conducted in accordance with the guidelines of Matsuyama University (protocol # 18-008). Nine-week-old male C57BL/6N mice were purchased from Japan SLC (Hamamatsu, Japan). Mice were kept at 23 ± 1 °C with a light/dark cycle of 12 h (light period 8:00–20:00, dark period 20:00–8:00). Animals were kept with free access to water and food for the duration of the experiment.

### 2.2. Sample Treatment

The control group (CON) was fed an 18% protein control diet, the protein malnutrition (PM) group a 2.5% low-protein diet, and the PM–CK group a PM diet mixed with 1% dried *C. kawachiensis* (CK) peel powder for the four-week feeding period. Then, the PM–LPS and PM–LPS–CK groups were intraperitoneally administered lipopolysaccharide (LPS) once (1 mg/kg of mouse), and mice were dissected either 2 or 8 days later. The group not administered LPS was treated with saline.

### 2.3. Food Intake

Mice were housed four per cage, and food consumption was measured each week. The amount of food consumed by one mouse was estimated by dividing the total food intake each day by the number of mice.

### 2.4. The Y-maze Test

Mice were placed at the tip of one of three arms (each arm was designated as A, B, or C) of a Y-shaped Y-maze device (length of 35 cm/width of 8.5 cm/height of 15 cm) and allowed to freely explore for 8 min. Mouse behavior was analyzed for the total distance travelled and immobility time using the ANY-maze Video Tracking System (Stoelting, Wood Dale, IL, USA) connected to a USB digital camera. A behavioral analysis was performed in a dark place with indirect illumination, feces and urine were processed for each test, and the apparatus was disinfected with alcohol each time to keep the inside clean.

### 2.5. Dissection

Blood was collected from the heart under anesthesia and followed by the transcardial perfusion of heparinized phosphate-buffered saline. The brain was removed and halved according to the cerebral longitudinal fissure. One half of the brain was soaked in 4% of paraformaldehyde for 2 days and then in 15 and 30% sucrose solutions for 1 day each. Brains were embedded in OCT compound, and 30 µm thick frozen brain sections (sagittal plane) were prepared using a cryostat (CM3050; Leica Microsystems, Heidelberger, Germany). The liver and gastrocnemius muscle were also collected. Blood samples were left to stand at room temperature for 30 min and then centrifuged at 1200× *g* at 4 °C for 20 min.

### 2.6. Blood Glucose Measurement

LabAssay™ Glucose (Mutarotase-GOD method; Wako, Osaka, Japan) was used to measure blood glucose levels. The experimental procedure was performed using the protocol in this kit, which involved measuring the absorbance of the sample and standard solutions at 570 nm with a microplate reader (Thermo Fisher, Tokyo, Japan).

### 2.7. Immunohistochemistry for Optical Microscopy

Thirty-micrometer-thick brain sections were immersed in 3% hydrogen peroxide solution for 20 min to remove endogenous peroxidase and then blocked in 2% skim milk for 60 min followed by 5% normal goat serum solution for 60 min. Sections were incubated with a rabbit polyclonal antibody against ionized calcium-binding adaptor 1 (Iba1, 1:1000; Wako, Osaka, Japan), which is specifically expressed in microglia, with shaking at 4 °C overnight. Brain sections were reacted with an Envision-plus system-HRP-labeled polymer as a secondary antibody for 60 min. The DAB substrate was used to develop staining, followed by 95% ethanol, 100% ethanol, and xylene × 2 in that order, and then sealed with a cover glass. Images of stained brain sections were captured with an optical microscope (CX21; Olympus, Tokyo, Japan). Multiple brain sections from each mouse were stained.

### 2.8. Immunofluorescence for Confocal Microscopy

Thirty-micrometer-thick brain sections were immersed in HistoVT One (Nacalai Tesque, Kyoto, Japan) and heated at 70 °C for antigen retrieval. Sections were then cooled to room temperature and blocked with 2% skim milk for 30 min followed by 5% normal goat serum solution for 60 min. Sections were incubated with a mouse monoclonal antibody against a synaptic vesicle membrane protein (synaptophysin, 1:1000; Sigma-Aldrich, St. Louis, MO, USA) as the primary antibody with shaking at 4 °C overnight. The next day, sections were reacted with Alexa Fluor 488 goat anti-mouse IgG (H + L) (1:300; Invitrogen, Carlsbad, CA, USA) as a secondary antibody and shaken for 60 min in the dark. Brain sections were placed on glass slides using mounting medium with DAPI (Vectashield; Vector Laboratories, Burlingame, CA, USA). Stained brain sections were then observed, and images were captured using a confocal fluorescence microscopy system (LSM800, Zeiss, Oberkochen, Germany). Multiple brain sections from each mouse were stained.

### 2.9. Image Analysis

A quantitative analysis of immune-positive signals in the images was performed using ImageJ software (NIH, Bethesda, MD, USA). The particle analysis tool was used for the analysis, and the pixels of the immune-positive signals were accumulated.

### 2.10. Statistical Analysis

Data for individual groups are expressed as means ± SEM. Data were statistically analyzed between two groups using the *t*-test. A value of *p* < 0.05 was significant.

## 3. Results

### 3.1. Changes in Body Weight and Food Intake in Different Experimental Groups

**Figure 1:**
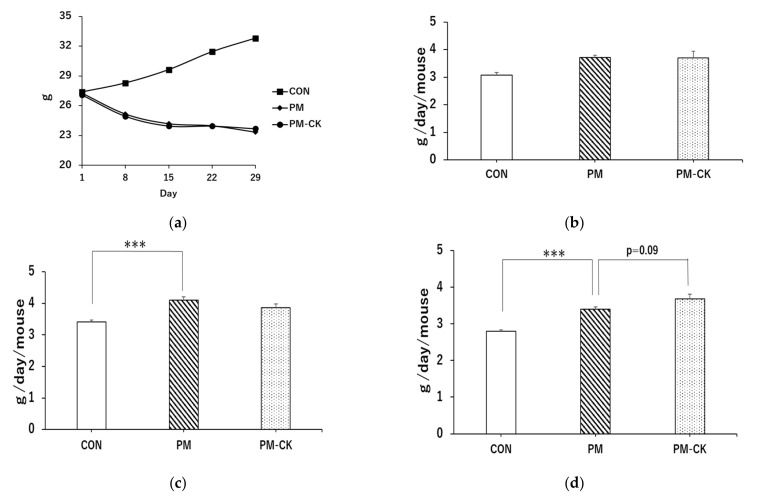
Body weight changes based on different experimental diets and average food intake per mouse before the administration of LPS. (**a**) Body weight changes during the experimental diet feeding period. The number of mice in each group is as follows: CON: *n* = 32, PM: *n* = 32, PM–CK: *n* = 32. Food intake in the (**b**) second, (**c**) third, and (**d**) fourth week after starting the experimental diet. Values are means ± SEM. Symbols show significant differences for the following conditions: CON vs. PM (*** *p* < 0.001). The number of samples in each group is as follows: CON: *n* = 4, PM: *n* = 4, PM–CK: *n* = 4.

Body weight showed normal increases in the CON diet group but decreased in the PM group (Figure 1a). However, food intake was significantly higher in the PM group than in the CON group (Figure 1b–d; *** *p* < 0.001). Furthermore, a comparison of food intake in the fourth week showed that intake was slightly higher in the PM–CK group than in the PM group (Figure 1d; *p* < 0.09).

### 3.2. Percent Weight Loss 2 or 8 Days after the Administration of LPS

**Figure 2:**
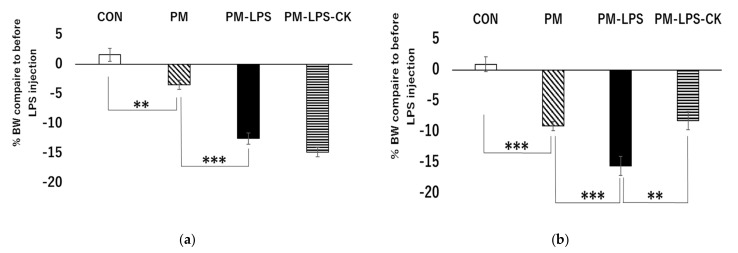
Body weight changes after the administration of LPS. The ratio of body weight changes (**a**) 2 days and (**b**) 8 days after the administration of LPS. Values are means ± SEM. Symbols show significant differences for the following conditions: CON vs. PM (** *p* < 0.01, *** *p* < 0.001). PM vs. PM–LPS (*** *p* < 0.001), and PM–LPS vs. PM–LPS–CK (** *p* < 0.01). The number of mice in each group is as follows: (**a**) CON: *n* = 8, PM: *n* = 8, PM–LPS: *n* = 7, PM–LPS–CK: *n* = 8, and (**b**) CON: *n* = 8, PM: *n* = 8, PM–LPS: *n* = 7, PM–LPS–CK: *n* = 7.

Comparisons of the CON and PM groups treated with saline showed that weight loss was significantly greater in the PM group than in the CON group 2 and 8 days after the administration of LPS (Figure 2a,b; ** *p* < 0.01, *** *p* < 0.001). Two days after the administration of LPS, weight loss was significantly greater in the PM–LPS group than in the PM group (Figure 2a; *** *p* < 0.001) and was not ameliorated by the dried *C. kawachiensis* peel powder (Figure 2a). Eight days after the administration of LPS, weight loss was significantly greater in the PM–LPS group than in the PM group (Figure 2b; *** *p* < 0.001). Body weight loss was significantly lower in the PM–LPS–CK group than in the PM–LPS group (Figure 2b; ** *p* < 0.01).

### 3.3. Ameliorative Effects of C. kawachiensis on Peripheral Dysfunction 8 Days after the Administration of LPS

**Figure 3:**
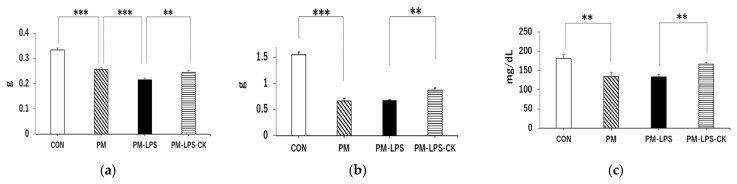
Effects of dried *C. kawachiensis* peel powder on protein malnutrition or LPS-induced peripheral dysfunction. Comparison of collected (**a**) gastrocnemius weight, (**b**) liver weight, and (**c**) blood glucose concentration in the 8-day group. Values are means ± SEM. Symbols show significant differences for the following conditions: CON vs. PM (*** *p* < 0.001, ** *p* < 0.01), PM vs. PM–LPS (*** *p* < 0.001), and PM–LPS vs. PM–LPS–CK (** *p* < 0.01, *** *p* < 0.001). The number of mice in each group is as follows: CON: *n* = 8, PM: *n* = 8, PM–LPS: *n* = 7, PM–LPS–CK: *n* = 7.

We collected gastrocnemius muscle, liver, and serum samples to examine the peripheral effects of the PM diet and inflammation. Gastrocnemius muscle weight was significantly lower in the PM group than in the CON group (Figure 3a; *** *p* < 0.001) and significantly lower in the PM–LPS group than in the PM group (Figure 3a; *** *p* < 0.001). However, gastrocnemius muscle weight loss was significantly lower in the PM–LPS–CK group than in the PM–LPS group (Figure 3a; ** *p* < 0.01). Liver weight was significantly lower in the PM group than in the CON group (Figure 3b; *** *p* < 0.001) but was similar in the PM–LPS group and PM group. On the other hand, liver weight loss was significantly lower in the PM–LPS–CK group than in the PM–LPS group (Figure 3b, ** *p* < 0.01). Blood glucose levels were also significantly lower in the PM group than in the CON group (Figure 3c; *** *p* < 0.001). Furthermore, the decrease observed in the PM–LPS group was significantly lower in the PM–LPS–CK group (Figure 3c; *** *p* < 0.001).

### 3.4. Ameliorative Effects of C. kawachiensis on Spontaneous Behavior

**Figure 4:**
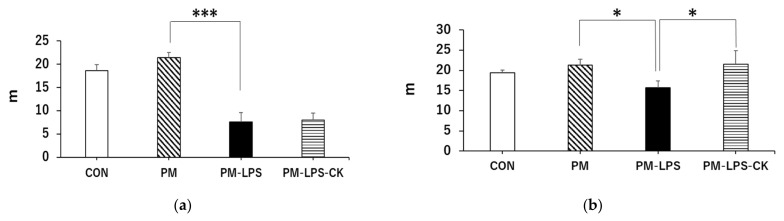

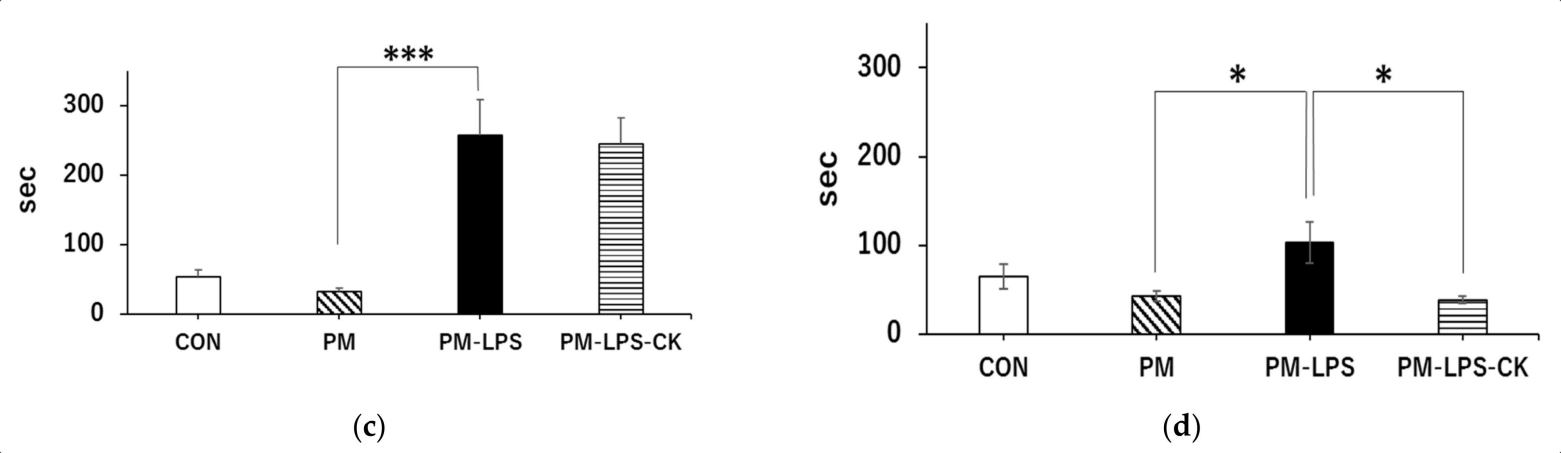
Effects of dried *C. kawachiensis* peel powder in the behavioral test. Measurement of total distance traveled in the (**a**) 2-day or (**b**) 8-day group in the Y-maze device. Measurement of immobility time in the (**c**) 2-day or (**d**) 8-day group using the ANY-maze Video Tracking System. Values are means ± SEM. Symbols show significant differences for the following conditions: PM vs. PM–LPS (* *p* < 0.05, *** *p* < 0.001), and PM–LPS vs. PM–LPS–CK (* *p* < 0.05). The number of mice in each group is as follows: (**a**,**c**) CON: *n* = 8, PM: *n* = 8, PM–LPS: *n* = 7, PM–LPS–CK: *n* = 8, and (**b**,**d**) CON: *n* = 8, PM: *n* = 8, PM–LPS: *n* = 7, PM–LPS–CK: *n* = 7.

We performed the Y-maze test to assess behavioral activity. The total distance traveled in 8 min did not significantly differ between the CON and PM groups 2 days after the administration of LPS (Figure 4a); however, it was significantly shorter in the PM–LPS group than in the PM group (Figure 4a; *** *p* < 0.001). Additionally, reductions in the total distance traveled were not attenuated by the dried *C. kawachiensis* peel powder. No significant differences were observed in immobility times 2 days after the administration of LPS between the CON and PM groups (Figure 4c), whereas it was significantly longer in the PM–LPS group than in the PM group (Figure 4c; *** *p* < 0.001). In addition, the immobility time in the PM–LPS–CK group was not suppressed (Figure 4c), as in the previous results. Eight days after the administration of LPS, no significant differences were noted in the total distance traveled between the CON and PM groups (Figure 4b); however, it was significantly shorter in the PM–LPS group than in the PM group (Figure 4b; * *p* < 0.05). The reduction observed in the total distance travelled in the PM–LPS group was significantly smaller in the PM–LPS–CK group (Figure 4b; * *p* < 0.05). Furthermore, no significant differences were noted in immobility times between the CON and PM groups (Figure 4d), whereas they were significantly longer in the PM–LPS group than in the PM group (Figure 4d; * *p* < 0.05). The longer immobility time observed in the PM–LPS group was reduced in the PM–LPS–CK group (Figure 4d; * *p* < 0.05).

### 3.5. Suppressive Effects of C. kawachiensis on Microglial Activation

**Figure 5:**
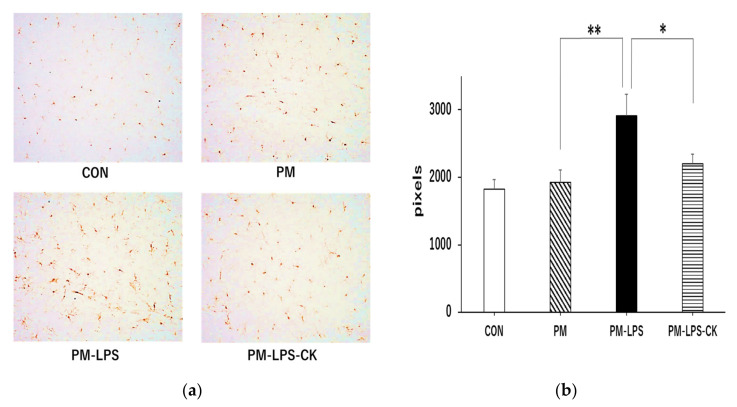
Effects of dried *C. kawachiensis* peel powder on microglial activity in the hippocampus of the 2-day group. (**a**) Representative microglia pictures stained with anti-Iba1 antibody. (**b**) Quantitative analysis of the area of Iba1-positive signals using ImageJ software. Values are means ± SEM. Symbols show significant differences for the following conditions: PM vs. PM–LPS (** *p* < 0.01), and PM–LPS vs. PM–LPS–CK (* *p* < 0.05). The number of samples in each group is as follows: CON: *n* = 23, PM: *n* = 23, PM–LPS: *n* = 23, PM–LPS–CK: *n* = 23.

The quantification of microglia in the stratum lacunosum-moleculare of the hippocampus revealed no significant differences between the CON and PM groups (Figure 5a,b); however, the total area of activated microglia was significantly higher in the PM–LPS group than in the PM group (Figure 5b; ** *p* < 0.01). This increase was significantly smaller in the PM–LPS–CK group (Figure 5b; * *p* < 0.05).

### 3.6. Protective Effects of C. kawachiensis on Neuronal Cell Function in the Hippocampus

**Figure 6:**
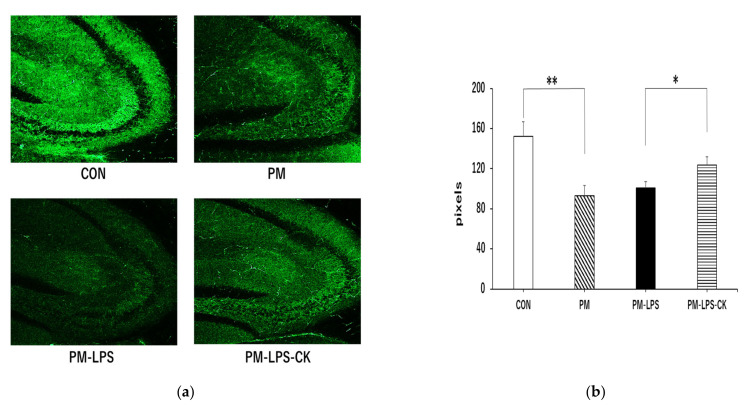
Effects of dried *C. kawachiensis* peel powder on synaptophysin immunoreactivity in the hippocampus of the 8-day group. (**a**) Representative synaptophysin antibody pictures stained with anti-synaptophysin antibody. (**b**) Quantitative analysis of the area of synaptophysin-positive signals using ImageJ software. Values are means ± SEM. Symbols show significant differences for the following conditions: CON vs. PM (** *p* < 0.01), and PM–LPS vs. PM–LPS–CK (* *p* < 0.05). The number of samples in each group is as follows: CON: *n* = 12, PM: *n* = 12, PM–LPS: *n* = 11, PM–LPS–CK: *n* = 10.

Synaptophysin as an indicator of synaptic vesicle membrane proteins in neurons was examined and quantified in the stratum lucidum of the hippocampal CA3 region. As shown in Figure 6, a significantly stronger synaptophysin signal was observed in the CON group than in the PM group (Figure 6a,b; *** *p* < 0.001). Although there was no significant difference between the PM and PM–LPS groups (Figure 6b), synaptophysin signaling was significantly stronger in the PM–LPS–CK group than in the PM–LPS group (Figure 6a,b; * *p* < 0.05).

## 4. Discussion

The peel of *C. kawachiensis* has been shown to exert protective effects on the brains of various pathological mouse models, including global cerebral ischemia, type 2 diabetes, and senescence-accelerated models, through anti-inflammatory, antioxidant, and neuroprotective activities [10,12,13,14]. HPLC and NMR analyses have revealed that the peel of *C. kawachiensis* contains larger amounts of bioactive components, such as HMF, AUR, and NGI, than other citrus varieties [11]. A previous study reported that the dried peel of *C. kawachiensis* contained 0.27 mg/g of HMF, 4.07 mg/g of AUR, and 44.02 mg/kg of NGI [10]. Furthermore, these components exert various effects. HMF exhibits anti-inflammatory activity, inhibits neuronal cell death, promotes BDNF production, stimulates neurogenesis, improves memory impairment, and exerts antidepressant effects [18,19,20,21,22,23,24]; AUR exhibits anti-inflammatory activity, inhibits neuronal cell death, promotes neurogenesis, and suppresses tau phosphorylation [25,26,27,28,29]. NGI also exhibits anti-inflammatory activity, inhibits neuronal cell death, promotes neurogenesis, suppresses tau phosphorylation, and exerts antioxidant effects [28,29,30].

Frailty is the result of the interplay of various factors, including chronic inflammation and oxidative stress associated with aging, which reduces physiological reserve capacity and increases vulnerability to stress [1,2,3]. Many patients with frailty have a poor nutritional status, particularly protein-energy malnutrition. These factors contribute not only to sarcopenia and locomotive syndrome, but also to diseases of the central nervous system, such as cognitive dysfunction and mental disorders. However, frailty is a reversible disease because improvements in any of these factors may lead to recovery; therefore, appropriate interventions are needed [1,2,3]. Protein malnutrition alters various parameters of oxidative stress [15,16]; furthermore, inflammation caused by LPS administration aggravates the pathology and produces a frailty model. This may replicate the chronic disease conditions in the body of the elderly.

In the present study, food intake was slightly higher in the group fed dried *C. kawachiensis* peel than in the PM group (Figure 1d). These results are consistent with the effects of “Chinpi”, the dried peel of *C. unshiu*, an herbal medicine with stomachic effects. Chinpi is a component of the Kampo medicines Rikkunshito and Ninjin’yoeito, both of which enhance digestive function and are effective against anorexia. Previous studies suggested that the flavonoids present in citrus peels increase the level of ghrelin, an appetite-stimulating hormone [31,32,33]. Furthermore, the peel of *C. unshiu* contains HMF, which enhances appetite through 5-HT_2_ receptor antagonism, and HMF in dried *C. kawachiensis* peel powder has also been suggested to increase food intake [34]. Various mechanisms for age-related sarcopenia have been reported, including anorexia, a decrease in anabolic hormones such as growth hormone and insulin-like growth factor, and damage caused by inflammatory cytokines [35]. An increased food intake may ameliorate the losses in liver and muscle weights caused by a PM diet, and the HMF-induced increase in ghrelin levels also stimulates growth hormone secretion via the growth hormone secretagogue receptor [32,33,36]. Ghrelin has also been reported to exert anabolic effects on protein and suppress inflammatory cytokines [32,35,37], and these combined effects may have prevented muscle and liver weight losses in the PM–LPS–CK group (Figure 3a,b). Blood glucose levels are maintained by glycogenolysis and glycogenesis in the liver even during fasting. In the present study, endogenous glucose production may have been reduced in the PM group due to a decrease in glycogen storage, a smaller liver volume, and a lack of the amino acids needed for glycogenesis [38,39]. In contrast, the repair of peripheral tissue function by *C. kawachiensis* as described above may have increased blood glucose levels (Figure 3c).

In the behavioral analysis, inflammation caused by LPS decreased activity 2 days after its administration, which may have been due to sickness behavior during the acute phase of inflammation (Figure 4a,c), consistent with previous findings [40]. The decrease observed in behavior in the PM–LPS group 8 days after the administration of LPS was attributed to the previously reported spread of inflammation from the periphery to the brain, which led to brain dysfunction and depression-like symptoms [40,41]. On the other hand, muscle and brain improvements associated with anti-inflammatory, antioxidant, neuroprotective, and anti-depressive effects in peripheral tissues and the central nervous system in the PM–LPS–CK group may have contributed to the recovery of behavioral activity (Figure 4b,d, Figure 5 and Figure 6) [12,14,19]. In the brain tissue analysis, the quantification of microglia in the acute inflammatory phase showed no significant inflammation in the PM group; however, inflammation was confirmed after the administration of LPS. This result also demonstrated that peripheral inflammation propagates to the central nervous system [40,41]; however, the administration of the dried peel of *C. kawachiensis* significantly suppressed microglial activation in the brain, which is consistent with previous findings on the effects of the peel in the brain (Figure 5a,b) [12,14]. In addition, the quantification of synaptic vesicle membrane proteins, a marker of neuronal cell function, showed low immunoreactivity signals in the PM group. In spite of our expectations, no further deterioration was observed with the administration of LPS. These results suggest that malnutrition due to protein deficiency alone may induce significant damage to the central nervous system, which is regarded as a major factor accelerating frailty [1,2,3,42]. However, the dried peel of *C. kawachiensis* significantly inhibited neuronal cell dysfunction induced by the PM diet and administration of LPS (Figure 6a,b), suggesting that it maintained brain function.

Nobiletin, which is structurally similar to HMF and a polymethoxyflavone found in *C. unshiu* and *C. depressa*, was recently shown to ameliorate memory impairment by reducing Aβ in the brain of animal models of Alzheimer’s disease and has anti-inflammatory and antioxidant effects [43,44]. Furthermore, AUR was found to exert antidepressant effects by lowering malondialdehyde (MDA) and nitrite concentrations in the brain, thereby enhancing antioxidant capacity [27]. Furthermore, NGI suppressed cognitive impairment and oxidative stress by reducing MDA and glutathione levels in the brain [30]. It has been also reported that many polyphenols as ingredients of citrus and other plants, including naringin, could increase PPAR-gamma, which works as an antioxidant [28,45]. The anti-inflammatory, antioxidant, and neuroprotective effects of dried *C. kawachiensis* peel powder, which is rich in these bioactive components, have been confirmed and other effects are expected [12,13,14].

## 5. Conclusions

Dried *C. kawachiensis* peel powder has the potential to attenuate peripheral tissue and central nervous system dysfunctions under frailty-like conditions caused by protein-deficient malnutrition. Furthermore, it may be an effective food material for physical and psychosomatic frailty. We have confirmed the effect of the dried peel of *C. kawachiensis* in various disease model mice, and we were able to confirm the effect of the dried peel of *C. kawachiensis* in this frailty-like model as well.

## Data Availability

Data is contained within the article.
